# Predictors of Serum Dioxins and PCBs among Peripubertal Russian Boys

**DOI:** 10.1289/ehp.0800223

**Published:** 2009-05-14

**Authors:** Jane S. Burns, Paige L. Williams, Oleg Sergeyev, Susan Korrick, Mary M. Lee, Boris Revich, Larisa Altshul, Donald G. Patterson, Wayman E. Turner, Larry L. Needham, Igor Saharov, Russ Hauser

**Affiliations:** 1 Environmental and Occupational Medicine and Epidemiology Program, Department of Environmental Health and; 2 Department of Biostatistics, Harvard School of Public Health, Boston, Massachusetts, USA; 3 Samara State Medical University, Department of Physical Education and Health, Samara, Russia; 4 Chapaevsk Medical Association, Chapaevsk, Samara Region, Russia; 5 Channing Laboratory, Department of Medicine, Brigham and Women’s Hospital, Harvard Medical School, Boston, Massachusetts, USA; 6 Pediatric Endocrine Division, Department of Pediatrics and Cell Biology, University of Massachusetts Medical School, Worcester, Massachusetts, USA; 7 Centers for Demography and Human Ecology of Institute for Forecasting, Russian Academy of Sciences, Moscow, Russia; 8 Exposure, Epidemiology, and Risk Program, Department of Environmental Health, Harvard School of Public Health, Boston, Massachusetts, USA; 9 EnviroSolutions Consulting, Inc., Jasper, Georgia, USA; 10 Centers for Disease Control and Prevention, Atlanta, Georgia, USA; 11 Ecological Analytical Center, Moscow, Russia

**Keywords:** children, diet, environment, epidemiology, polychlorinated biphenyls, polychlorinated dibenzodioxins, polychlorinated dibenzofurans

## Abstract

**Background:**

Although sources and routes of exposure to dioxins and polychlorinated biphenyls (PCBs) have been studied, information regarding exposure among children is limited. Breast-feeding and diet are two important contributors to early life exposure. To further understand other significant contributors to childhood exposure, we studied a cohort of children from a city with high environmental dioxin levels.

**Objectives:**

We investigated predictors of serum concentrations of polychlorinated dibenzo-*p*-dioxins (PCDDs)/polychlorinated dibenzofurans (PCDFs)/co-planar PCBs (C-PCBs), toxic equivalents (TEQs), and PCBs among 8- to 9-year-old boys in Chapaevsk, Russia.

**Methods:**

We used general linear regression models to explore associations of log_10_-transformed serum concentrations of PCDDs/PCDFs/C-PCBs, TEQs, and PCBs at study entry with anthropometric, demographic, geographic, and dietary factors in 482 boys in Chapaevsk, Russia.

**Results:**

The median (25th, 75th percentile) concentration for total 2005 TEQs was 21.1 pg/g lipid (14.4, 33.2). Boys who were older, consumed local foods, were breast-fed longer, and whose mothers were employed at the Khimprom chemical plant (where chlorinated chemicals were produced) or gardened locally had significantly higher serum dioxins and PCBs, whereas boys with higher body mass index or more educated parents had significantly lower serum dioxins and PCBs. Boys who lived < 2 km from Khimprom had higher total TEQs (picograms per gram lipid) [adjusted mean = 30.6; 95% confidence interval (CI), 26.8–35.0] than boys who lived > 5 km away (adjusted mean = 18.8; 95% CI, 17.2–20.6).

**Conclusions:**

Our findings suggest that there are specific local sources of dioxin and PCB exposure among children in Chapaevsk including maternal gardening, consumption of locally grown food, and residential proximity to the Khimprom plant.

Environmental exposure to dioxins [polychlorinated dibenzo-*p*-dioxins (PCDDs)], dioxin-like compounds [polychlorinated dibenzofurans (PCDFs) and co-planar polychlorinated biphenyls (C-PCBs)], and polychlorinated biphenyls (PCBs) has been associated with an increased risk of immune dysfunction (Nagayama et al. 2007), cancer (Consonni et al. 2008; Michalek and Pavuk 2008; Steenland et al. 1999), adverse cardiovascular effects (Humblet et al. 2008), reproductive and developmental effects (Mocarelli et al. 2008; Wigle et al. 2008; Wolff et al. 2008), hormone disruption (Baccarelli et al. 2008; Maervoet et al. 2007), and diabetes mellitus (Consonni et al. 2008; Longnecker and Michalek 2000; Wang et al. 2008). These environmental contaminants were produced as by-products of industrial processes and incineration or released into the environment consequent to their past industrial use. These compounds are slow to degrade, lipophilic, and have a long half-life, with the body burden increasing with age. In the general population, diet is the most significant source of exposure, with breast-feeding transferring a portion of the mother’s body stores of these compounds to her infant (Fürst 2006).

Children are especially vulnerable to dietary exposure to dioxins and PCBs because of their high food consumption relative to their body weight (Schecter et al. 2001). Dermal absorption, inhalation, and hand-to-mouth transfer from contaminated dust and soil are additional exposure routes of particular concern for children. Because these compounds degrade slowly in soil and aquatic sediment, children may be more highly exposed because they spend more time engaged in outdoor activities than adults, especially if they live near contaminated areas (Hauser et al. 2005; Petrik et al. 2006). Mothers with occupational exposure to dioxins or PCBs may also be a source of exposure to their children via transference of the higher body burden to the children through breast-feeding (Patandin et al. 1999). Breast-fed children, compared with formula-fed children, have higher serum levels of these compounds (Abraham et al. 1996). Although pharmacokinetic models have indicated that serum dioxin levels of breast-fed children should decline to converge with formula-fed children by ≤ 7 years of age (Kerger et al. 2007; Kreuzer et al. 1997), prospective sampling has indicated that levels remain higher as late as 10 years after birth (Heudorf et al. 2002; Link et al. 2005). However, few studies have assessed the contribution of multiple potential sources of dioxin and PCB exposure to children’s blood levels of these compounds.

We studied predictors of dioxin exposure among children residing in a community with high environmental contamination of dioxins, furans, and PCBs, specifically in Chapaevsk, Russia. There had been an extensive chemical manufacturing industry in Chapaevsk since the 1930s. Since 1949, the Khimprom chemical plant has produced chlorine-containing industrial and agricultural chemicals such as γ-hexachlorocyclohexane, liquid chlorine, dichloropropionic acid, hexachlorobenzene (HCB), methyl chloroform, vinyl chloride, and pentachlorophenol (Ecological Analytical Center 2007; Revich et al. 1996). As a byproduct of the manufacturing process and waste incineration (Shelepchikov et al. 2008), there has been widespread local environmental and food contamination by PCDDs and PCDFs, resulting in higher levels compared with other regions in Russia (Revich et al. 1999, 2001; Sergeyev et al. 2007). Previous studies have documented elevated levels of these compounds in the local population in serum (Akhmedkhanov et al. 2002; Hauser et al. 2005) and breast milk (Sergeyev et al. 2008) compared with non-occupationally exposed populations in other parts of Russia, Europe, and the United States.

The children in the present report are participants in the ongoing Russian Children’s Study of the effect of dioxins and PCBs on male growth and sexual maturation in Chapaevsk. This study was initiated in response to concerns of Russian colleagues about possible increased rates of congenital reproductive-tract anomalies in Chapaevsk boys. To identify predictors of serum dioxin and PCB levels, we measured serum dioxin and PCB concentrations and collected extensive information on sources of potential exposures to these compounds.

## Methods

### Study population

The Russian Children’s Study is a prospective cohort study of 499 peripubertal boys in Chapaevsk, Russia. The 8- and 9-year-old boys were identified using the townwide health insurance information system and were enrolled from 2003 to 2005. Children were excluded if they were institutionalized (e.g., living in orphanages), because of missing birth or family history information, because of Azerbaijani nationality (they were likely to relocate during the study period), or had a chronic illness that could affect childhood growth and development.

The study was approved by the Human Studies Institutional Review Boards of the Chapaevsk Medical Association, Harvard School of Public Health, University of Massachusetts Medical School, and Brigham and Women’s Hospital. The parent or guardian signed an informed consent, and the boy signed an assent form.

After consenting to participate, eligible boys had a physical examination and provided blood samples for the analyses of dioxins and PCBs. Along with their mothers or guardians, they completed nurse-administered health, lifestyle, and dietary questionnaires.

### Health and lifestyle questionnaires

The health and lifestyle questionnaires were developed through pilot work in cooperation with our Russian collaborators in Chapaevsk (Hauser et al. 2005; Lee et al. 2003). Information on age, birth history (birth weight, prematurity), history of breast-feeding, family medical, occupational, and residential history, household income, parental education, lifestyle factors such as smoking and alcohol use, and the child’s medical history and physical activity was collected.

### Physical examination and blood samples

At the initial study visit, a standardized anthropometric examination was performed by a single study investigator (O.S.) according to a written protocol and without knowledge of the child’s residential location. Height in stocking feet was measured to the nearest 0.1 cm, using a stadiometer. Weight was measured to the nearest gram with a metric scale. Body mass index (BMI; kilograms per square meter) was calculated from the weight (kilograms) and height (meters) measurements.

Blood samples were collected before the examination. Samples were centrifuged and the serum was aliquoted and stored at −35°C until shipment on dry ice to the Centers for Disease Control and Prevention (CDC) for analysis. The chemical analyses were performed by the National Center for Environmental Health, CDC, Atlanta, Georgia. Serum samples were spiked with a mixture of ^13^C_12_-labeled PCDDs/PCDFs and C-PCBs as internal standards, and the analytes were isolated from serum by a C_18_ solid phase extraction (SPE) followed by a multicolumn automated cleanup and enrichment procedure (Turner et al. 1997). Samples were processed in batches of 10, which included a method blank and two quality control samples that were aliquots of pooled bovine sera spiked with PCDDs, PCDFs, and C-PCBs.

The analytes were separated on a DB-5 MS capillary column [Agilent JW Scientific DB-5ms (p/n 122-5532); Agilent Technologies, Santa Clara, CA] and quantified using selected-ion-monitoring, high-resolution (10,000 resolving power) mass spectrometry (Patterson et al. 1987).

Quantification was by isotope dilution mass spectrometry using calibration standards containing ^13^C- labeled and -unlabeled analytes. For specific PCDDs, PCDFs, and C-PCBs that lack their own labeled standard, a labeled congener with the same degree of substitution and a similar retention time was used. Mono-*ortho* PCBs (M-PCBs) and non-dioxin-like PCBs were extracted from an aliquot (1 g) of sample by SPE extraction (Turner et al. 1997). The serum lipid content was determined from enzymatic measurements of total cholesterol and triglycerides (Phillips et al. 1989).

### Dietary information

A validated Russian Institute of Nutrition (RIN) semiquantitative food frequency questionnaire (FFQ) (Martinchik et al. 1998) was modified to ascertain the child’s typical dietary intake over the previous year (Rockett et al. 1997), including the consumption of locally grown or raised food, and was administered by a study nurse at the initial study visit. The FFQ contained questions on > 70 food items and used a picture book prepared by the RIN to assess portion size.

### Geographic information

We generated an electronic map (scale 1 cm:100 m) of Chapaevsk, with coordinates (*x*,*y*) for the families’ residences and the centers of the Khimprom chemical plant, workshop, and storage areas plotted on a plan of the town (by I.S.). Using ArcView GIS 3.0 (ESRI, Redlands, CA, USA), the straight line distances were calculated for each residence to the Khimprom plant centers.

### Statistical analysis

We analyzed the cross-sectional association of potential predictors with the boys’ serum dioxin and PCB concentrations measured at study entry. Serum samples below the limit of detection (LOD) were assigned a value equal to the LOD divided by the square root of 2 (Baccarelli et al. 2005). We grouped the dioxin and PCB congeners into summary measures: lipid-adjusted serum concentration of total PCDDs/PCDFs/C-PCBs, lipid-adjusted serum 1998 and 2005 toxic equivalents (TEQs), and lipid-adjusted serum concentration of total PCBs. These summary measures and the individual congeners were log_10_-transformed to improve normality. We used general linear regression models to assess associations of serum dioxins, furans, and PCBs with anthropometric, demographic, lifestyle, geographic, and dietary covariates. Initially we evaluated univariate associations, then fitted a full multivariate model including all covariates with *p* ≤ 0.20, and finally reduced to a core model that included covariates with *p* < 0.10. We also examined predictors of selected individual congeners and congener groupings. We examined the congeners 2,3,7,8-tetrachlorodibenzo-*p*-dioxin (TCDD), PCB 3,3′,4,4′,5 (PCB-126), and PCB 3,3′,4,4′ (PCB-77) because of either their contribution to serum total TEQs or prior literature linking them to health effects (Arsenescu et al. 2008; Mocarelli et al. 2008). We also examined separately the groups of PCDDs with more than a 5% contribution to the total TEQs [TCDD, 1,2,3,7,8-pentachlorodibenzo-*p*-dioxin (1,2,3,7,8-PeCDD), 1,2,3,6,7,8-hexachlorod-ibenzo-*p*-dioxin (1,2,3,6,7,8-HxCDD)] and PCDFs that were produced during chlorine production or incineration [1,2,3,4,6,7,8-heptachlorodibenzo furan (1,2,3,4,6,7,8-Hp-CDF), 1,2,3,4,7,8-hexachlorodibenzofuran (1,2,3,4,7,8-HxCDF), 1,2,3,6,7,8-hexachloro-dibenzofuran (1,2,3,6,7,8-HxCDF)]. Because some congeners had a substantial percentage (> 25%) of serum concentrations below the LOD, we conducted a sensitivity analysis using generalized estimating equations, which are more robust to departures from normality. The results were very similar to the results of the linear regression models and were therefore not presented

Local food categories were eggs, dairy products, poultry, nonpoultry meat (pork, beef, and lamb products), fish, and fruits and vegetables. With the exception of fruit and vegetables, less than half of the boys reported eating local foods; thus, all other food categories were modeled as the consumption of any local food versus none. Observed local fruit and vegetable consumption clustered around five distinct intake levels (< 7.5, 7.6–85.8, 85.9–146.6, 146.7–229.6, > 229.7 kg/year), so intake was modeled as five ordered categories of consumption (in grams), comparing each of the higher levels separately with the lowest level.

**Figure 1:**
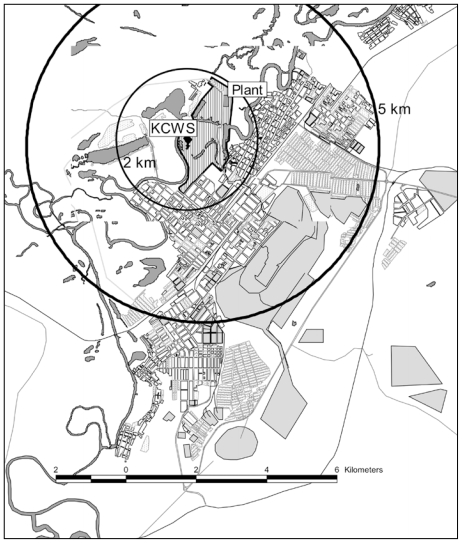
Map of Chapaevsk, location of Khimprom (plant), Khimprom chemical workshop (KCWS).

We first evaluated the associations of serum dioxin and PCB concentrations with quintiles of geographic distance of residence to the three chemical plant areas (i.e., center of plant, storage area, and workshop). Because the regression coefficients associated with adjacent quintiles for distances were very similar, in the final model the distances were categorized as < 2 km, 2–5 km, and > 5 km (Figure 1).

We used the core model of key anthropometric and demographic covariates and included each food category separately while adjusting for total food consumption (in grams). Final models included all anthropometric and demographic covariates with *p* < 0.10 in addition to the dietary (entered separately) and geographic covariates identified as significant predictors of serum dioxins or PCBs.

## Results

### Study population

Among the 499 boys, height, weight, and BMI (Table 1) were within the normal range according to the World Health Organization (WHO) Child Growth Standards (Cole et al. 2000, 2007; WHO 2007). Eighty-seven percent of mothers reported breast-feeding their sons for at least 1 month, with an average of 6 months’ duration. Approximately half of the parents lived in Chapaevsk for 6 years or more, and more than half of the mothers kept a local garden. Almost one-fifth of the families lived within 2 km of the Khimprom chemical plant, with 13% of fathers and only 6% of mothers reporting past employment at the plant.

### Serum dioxins and PCBs

Among the 482 boys who had dioxin measurements, there was a wide range of serum concentrations and corresponding WHO 2005 TEQs (Van den Berg et al. 2006) for dioxins, furans, and PCBs. The distributions were skewed; thus, the medians and percentiles are presented rather than means (Table 2). PCDDs had the largest contribution (38%) to the total 2005 TEQs, followed by C-PCBs (29%), PCDFs (25%), and the M-PCBs (8%). The congeners with the greatest individual contributions to the total 2005 TEQs were the C-PCB 3,3′,4,4′,5-PeCB (PCB-126) (26%), 1,2,3,7,8-PeCDD (19%), 2,3,4,7,8-PeCDF (16%), and TCDD (11%). In comparison, the total WHO 1998 TEQs median (25th, 75th percentiles) (29.4; 20.7, 46.4) was higher than the total 2005 TEQs median (25th, 75th percentiles) (21.1; 14.4, 33.2), primarily because of the higher toxic equivalency factor for the M-PCBs in the 1998 TEQ classification. Total serum PCB concentrations had a median (25th, 75th percentiles) of 249 ng/g lipid (164, 393). The largest individual congeners’ contributions were PCB-153 (23%), PCB-138-158 (20%), and PCB-99 (11%).

### Local food consumption

Most (92%) of the boys ate locally grown fruits and vegetables. Nearly half (46%) of the boys ate local dairy products. Approximately one-fifth (21%) of the boys ate locally caught fish, 16% ate locally produced eggs, 7% ate poultry, and 5% ate nonpoultry meats.

### General predictors of serum dioxins and PCBs

An older age and longer duration of breast-feeding were associated with significantly higher serum dioxins and PCBs, whereas higher BMI was associated with significantly lower serum dioxins and PCBs (Tables 3–5). Boys who were breast-fed for 26 weeks had a 28% increase in serum TEQs [adjusted mean = 24.5; 95% confidence interval (CI), 20.5–28.6] compared with no breast-feeding (adjusted mean = 19.1; 95% CI, 16.5–22.1). Boys whose mothers had a local garden had significantly higher serum dioxins (2005 TEQ adjusted mean: 25.4; 95% CI, 23.6–27.3) and PCBs compared with those who did not (2005 TEQ adjusted mean: 20.6; 95% CI, 18.9–22.4) (Tables 3–5). Boys whose mothers reported ever working at Khimprom had significantly higher serum dioxins and PCBs compared with those whose mothers never worked at Khimprom (Tables 3–5), although the associations with PCBs were marginally significant (Table 5). Higher maximum parental education was associated with significantly lower serum PCDD/PCDF/C-PCB concentrations and marginally significant lower 2005 TEQs, but not with lower PCBs (Tables 3–5). Longer residence in Chapaevsk was associated with higher serum 2005 TEQs and PCBs, but not with serum PCDDs/PCDFs/C-PCBs (Tables 3–5).

For the subanalyses, the associations of serum TCDD, PCB-126, and the subgroups of PCDD and PCDF congeners with age, breast-feeding, BMI, residence < 2 km from the Khimprom chemical plant, and consumption of local eggs and dairy were similar to the summary dioxin and PCB measures. Longer residence in Chapaevsk was significantly associated with TCDD, PCB-126, and PCDD subgroup. PCB-77 did not demonstrate the same pattern of associations as the summary dioxin and PCB measures except for significant associations with age and consumption of local eggs and dairy.

### Geographic distance and serum dioxins and PCBs

Serum dioxin and PCB concentrations of the boys were most strongly associated with distance to the Khimprom chemical plant workshop. Therefore, in Tables 3–5, we present only the results for associations with the workshop. Boys who lived < 2 km from the workshop had adjusted mean serum 2005 TEQs of 30.6 (95% CI, 26.8–35.0) compared with those who lived 2–5 km from the workshop (adjusted mean = 22.2; 95% CI, 20.7–23.8) or > 5 km from the workshop (adjusted mean serum = 18.8; 95% CI, 17.2–20.6), such that serum TEQ levels were 63% higher for boys who lived < 2 km versus those who lived > 5 km from the workshop (trend test *p* < 0.0001). The serum concentrations of PCDDs/PCDFs/C-PCBs, and total PCBs of boys living < 2 km from the workshop were 33% and 25% higher, respectively, than those who lived > 5 km away (trend test *p* < 0.0001 and *p* = 0.007, respectively).

### Local dietary intake and serum dioxins and PCBs

**Figure 2:**
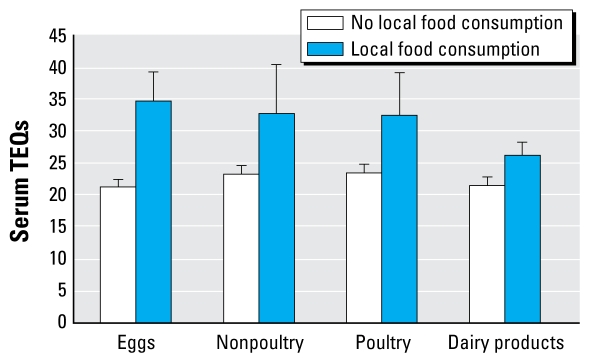
Mean serum 2005 TEQs by consumption of locally grown foods among 482 8- to 9-year-old boys in Chapaevsk, Russia (adjusted for age, BMI, breast-feeding, parental education, residence in Chapaevsk, mother’s employment at Khimprom, mother’s local gardening, residential distance from Khimprom).

Boys who consumed any local eggs, nonpoultry meats, poultry, or dairy had significantly higher serum concentrations of dioxins and PCBs (Tables 3–5) compared with boys who had no local consumption. Serum TEQs were higher, on average, for eggs (64%), nonpoultry and poultry meats (42%), and dairy (22%) compared with those who had no local consumption (Figure 2).

The consumption of any local fish compared with none was associated with significantly higher serum total TEQs and PCBs but not with higher serum PCDDs/PCDFs/C-PCBs (Tables 3–5). Serum total TEQs were significantly higher among boys with the highest category of local fruit and vegetable consumption compared with boys in the lowest category of local fruit and vegetable consumption (Table 4).

## Discussion

Our cohort of peripubertal boys in Chapaevsk, Russia, had high serum levels of dioxins and PCBs compared with other populations (Link et al. 2005; Patterson et al. 2008). Like other studies, we found that older age of the boy, longer breast-feeding duration, and consumption of local foods were associated with higher serum concentrations of dioxins and PCBs (Choi et al. 2006; Hauser et al. 2005; Link et al. 2005). Residential proximity to and maternal employment at Khimprom, as well as maternal local gardening, were associated with higher serum dioxins and PCBs among the boys.

Studies of serum dioxin and PCB concentrations among children are limited, and we are unaware of data from other Russian children. The median serum total 2005 TEQs of the 8- to 9-year-old Chapaevsk boys was triple the geometric mean from the U.S. National Health and Nutrition Examination Survey for males 12–19 years of age (there were no data on children < 12 years of age) (Patterson et al. 2008). Among this U.S. age group, PCDDs/PCDFs contributed 86% to the total TEQs compared with 63% of total TEQs in Chapaevsk boys, suggesting proportionately greater exposure to C-PCBs and M-PCBs among the boys in Chapaevsk. Similarly, serum total 1998 TEQs of the Chapaevsk children were almost triple the levels measured among German children (mean age, 10.2 years) during 2002–2003 (Link et al. 2005). However, the contribution of PCDDs/PCDFs to the total 1998 TEQs was comparable between the German (49%) and Chapaevsk (46%) children.

The results of our study suggest that industrial contamination of the local environment may be an important source of exposure for the Chapaevsk boys. Boys who lived closest to the Khimprom plant had significantly higher serum dioxin and PCB concentrations, and environmental (soil, house dust) and human (breast milk, serum) samples from residential areas closest to Khimprom had higher dioxin and PCB levels (Sergeyev et al. 2007, 2008). The finding of high serum PCBs was unexpected, because PCBs were not manufactured at Khimprom, although they may have been used at the plant. The summary measures based predominantly on PCBs were also higher among boys who had lived longer in Chapaevsk, even after adjustment for age, suggesting that the local environment and foods of Chapaevsk may be an important source of PCB exposure. The boys whose mothers reported having local gardens had higher serum dioxin and PCB levels, even after adjustment for local food consumption. This suggests the possibility that mothers exposed to contaminated garden soil may have transferred these exposures to their sons by breast-feeding or that their sons were involved in gardening or playing in the garden area, thereby increasing their exposure to these compounds through contact with the contaminated garden soil.

Dietary consumption of local eggs, meats, poultry, dairy, and fish were significantly associated with higher serum dioxins and PCBs among the Chapaevsk boys. In other studies, elevated blood dioxin and PCB concentrations have been linked to local food consumption in areas with environmental exposures to these compounds (Choi et al. 2006; Hauser et al. 2005; Schecter et al. 2003). Research in Chapaevsk suggests that local eggs and fish have significantly higher concentrations of dioxins and PCBs compared with other regions in Russia (Sergeyev et al. 2007; Shelepchikov et al. 2006). Although the mechanism for egg contamination is unknown, it may be either the result of chickens feeding in contaminated soil or eating contaminated feed, as suggested in other studies (Hayward and Bolger 2005; Schoeters and Hoogenboom 2006). Local nonpoultry meat and poultry consumption were also associated with higher serum dioxin and PCB concentrations among the boys. Although prior studies have found that meats contribute to dioxin and PCB exposure (Charnley and Doull 2005; Fernandez et al. 2004), < 10% of the Chapaevsk boys ate meats from local sources; thus, this association should be cautiously interpreted. The associations between the consumption of local nonpoultry meat, poultry, their products such as eggs and dairy, and fish with higher serum dioxins and PCBs suggest that both local animals and their products may be important sources of these compounds. Animal products such as eggs, milk, and cheese are important sources of protein in Chapaevsk. This is enhanced by the local population having access to animal products from local farms; nearly half of the study participants reported eating local dairy products, and 16% ate local eggs. Additionally, most families in this community consume local fruits and vegetables, and the boys who consumed the most had significantly higher serum total TEQ concentrations. The observation that local foods were associated with higher serum dioxin and PCB concentrations presents a dilemma for this community, where the consumption of local foods is both a common practice and an important source of protein, fruits, and vegetables.

Boys whose mothers had been employed in the Khimprom plant had higher serum dioxin and PCB levels, suggesting these mothers may have had occupational exposure with increased body burdens and transferred more of these compounds to their sons via breast-feeding. Consistent with this finding, some of the children of workers in a phenoxy herbicide chemical plant in Ufa, Russia, had elevated serum TCDD concentrations (Ryan and Schecter 2000). However, because only 6% of the mothers were ever employed at the Khimprom plant, the association with higher serum dioxin and PCB concentrations among their sons should be interpreted cautiously.

In our study, occupation of the mothers and local gardening were associated with higher serum dioxin and PCBs levels among the sons. Although we carefully assessed and controlled for potential confounders of these associations, such as household income, parental education, residence, and dietary consumption of local foods, this adjustment may not have been sufficient.

Boys with higher BMIs had lower serum dioxin and PCB concentrations. Similarly, a cross-sectional analysis of children in Germany also found an inverse association between serum PCBs and body weight (Link et al. 2005). Typically, higher BMI for adults is associated with higher serum concentrations of these compounds (Bilau et al. 2009; Collins et al. 2007; Michalek et al. 1996). However, in a follow-up analysis of the Seveso cohort, neither BMI nor percent body fat was associated with serum dioxin concentrations after adjustment for sex (Landi et al. 1998). The metabolism of dioxins and PCBs between children and adults are dissimilar in some respects, with dioxin-like compounds having shorter half-lives among children compared with adults (Kerger et al. 2006). In children, these lipophilic compounds may be more likely to be sequestered in the body fat or, for those with higher BMI, increased growth may have resulted in dilution of dioxin-like compounds.

We investigated other measures of adiposity, such as waist circumference, waist-to-hip ratio, and clinical assessment of body fat, and found that the inverse associations between BMI and serum dioxin and PCB concentrations were consistent across these measures. Based on these preliminary findings, bioelectric impedance measurements have been added to the study to assess adiposity, so that future analyses may clarify the association between BMI and body composition with serum dioxins and PCBs in this cohort of boys.

Residence near or maternal occupation at the chemical plant, local gardening, and the consumption of local foods were predictors of higher serum dioxin and PCB concentrations among the boys. These data suggest that people who reside near a contaminated area may need to be aware of potential risk for exposure through contact with local soil or consumption of local foods. Although consumption of some local foods contributed to dioxin and PCB exposure, it is important to consider that these locally grown foods are also important components of a nutritious diet for the children of Chapaevsk. Recommendations to reduce children’s exposure include remediation of soil, which is ongoing in specific highly contaminated areas, or relocating gardens and plots to areas with lower contamination.

## Figures and Tables

**Table 1:** Descriptive characteristics of the Russian Children’s Study (participants).

Characteristic	8-year-old boys (*n* = 309)	9-year-old boys (*n* = 190)	Total boys (*n* = 499)
Boys (mean ± SD) by age group^a^			
Height (cm)	128.5 ± 6.0	133.1 ± 6.0	
Weight (kg)	26.6 ± 5.6	28.5 ± 5.8	
BMI (kg/m^2^)	16.0 ± 2.3	16.0 ± 2.4	
Duration of breast-feeding (weeks)	25.0 ± 33.8	31.8 ± 36.0	

Family [no. (%)]^a^			
Mother’s age at son’s birth ≤ 25 years			353 (71)
Son is firstborn child			319 (64)
Maximum parental education			
Secondary education or less			41 (8)
Junior college/technical training			293 (59)
University graduate			161 (33)
Household income (US$/month)			
< 175			178 (36)
175–250			127 (25)
> 250			193 (39)
Father ever employed at Khimprom			63 (13)
Mother ever employed at Khimprom			28 (6)
Residence in Chapaevsk ≥ 6 years			256 (51)
Mother’s local gardening			299 (60)
Current residence, distance from Khimprom			
< 2 km			95 (19)
2 to < 5 km			225 (45)
≥ 5 km			176 (36)

a Missing information: breast-fed (*n* = 12); mother’s age (*n* = 5); firstborn (*n* = 20); parental education (*n* = 4); household income (*n* = 1); father employed at Khimprom (*n* = 28); mother employed at Khimprom (*n* = 15); Chapaevsk residence (*n* = 2); mother’s local gardening (*n* = 10); current residence, distance from Khimprom (*n* = 3).

**Table 2:** Distribution of serum PCDDs/PCDFs/C-PCBs (pg/g lipid) concentrations and 2005 WHO TEQs among 8- to 9-year-old boys in the Russian Children’s Study (*n* = 482).

	Median LOD^b^	Samples below LOD (%)	Concentration^a^ percentiles			2005 WHO TEQs (pg TEQ/g lipid) percentiles		
			25th	Median	75th	25th	Median	75th
PCDDs (pg/g lipid)								
TCDD	0.60	26	1.34	2.75	3.90	1.34	2.75	3.90
1,2,3,7,8-PeCDD	0.70	28	1.41	4.10	7.00	1.41	4.10	7.00
1,2,3,4,7,8-HxCDD	1.10	63	0.71	2.00	3.90	0.07	0.20	0.39
1,2,3,6,7,8-HxCDD	1.10	12	5.40	8.70	16.6	0.54	0.87	1.66
1,2,3,7,8,9-HxCDD	1.10	52	0.85	2.61	4.60	0.09	0.26	0.46
1,2,3,4,6,7,8-HpCDD	1.20	< 1	8.20	12.2	19.5	0.08	0.12	0.20
OCDD	16.1	26	69.0	96.1	134	0.02	0.03	0.04
PCDFs (pg/g lipid)								
2,3,7,8-TCDF	0.70	91	0.42	0.50	1.63	0.04	0.05	0.16
1,2,3,7,8-PeCDF	0.70	83	0.42	0.57	1.91	0.01	0.02	0.06
2,3,4,7,8-PeCDF	0.60	3	6.20	9.0	14.6	1.86	2.70	4.38
1,2,3,4,7,8-HxCDF	0.70	2	4.10	6.65	12.5	0.41	0.67	1.25
1,2,3,6,7,8-HxCDF	0.70	11	2.90	4.20	6.70	0.29	0.42	0.67
1,2,3,7,8,9-HxCDF	0.80	99	0.42	0.57	1.41	0.04	0.06	0.14
2,3,4,6,7,8-HxCDF	0.70	88	0.42	0.57	1.84	0.04	0.06	0.18
1,2,3,4,6,7,8-HpCDF	0.80	7	5.47	7.50	11.3	0.06	0.08	0.11
1,2,3,4,7,8,9-HpCDF	0.80	85	0.50	0.64	2.26	0.01	0.01	0.02
OCDF	0.90	26	1.80	2.90	5.00	0.001	0.001	0.002
C-PCBs (pg/g lipid)								
3,3′,4,4′-TCB 77	1.40	0	48.9	88.1	134	0.005	0.009	0.01
3,4,4′,5-TCB 81	1.50	3	5.90	8.45	12.6	0.002	0.003	0.004
3,3′,4,4′,5-PeCB 126	1.50	< 1	40.3	58.0	84.4	4.03	5.80	8.44
3,3′,4,4′,5,5′-HxCB 169	1.30	2	11.1	16.9	28.9	0.33	0.51	0.87
M-PCBs (ng/g lipid)								
2,3,3′,4,4′-PeCB (105)	0.30	< 1	4.90	7.40	11.1	0.15	0.22	0.33
2,3′,4,4′,5-PeCB (118)	0.30	< 1	22.4	33.1	48.7	0.67	0.99	1.46
2,3,3′4,4′,5-HxCB (156)	0.40	1	3.60	5.80	10.7	0.11	0.17	0.32
2,3,3′,4,4′,5′-HxCB (157)	0.40	14	1.00	1.80	3.30	0.03	0.05	0.10
2,3′,4,4′,5,5′-HxCB (167)	0.50	11	1.30	2.10	3.45	0.04	0.06	0.10
2,3,3′,4,4′,5,5′-HpCB (189)	0.60	73	0.21	0.57	0.78	0.006	0.02	0.02
Total PCDDs (pg/g lipid)			93	136	189	4.5	8.2	13.5
Total PCDFs (pg/g lipid)			27	39	57	3.0	4.2	6.9
Total C-PCBs (pg/g lipid)			126	181	249	4.5	6.4	9.4
Total PCDD/F/C-PCBs (pg/g lipid)			278	362	499	13.3	19.6	30.5
Total M-PCBs (ng/g lipid)			35	52	78	1.1	1.6	2.4
Total PCBs (ng/g lipid)			164	249	393			
Total TEQs						14.4	21.1	33.2

a Samples below the LOD were assigned a value = 
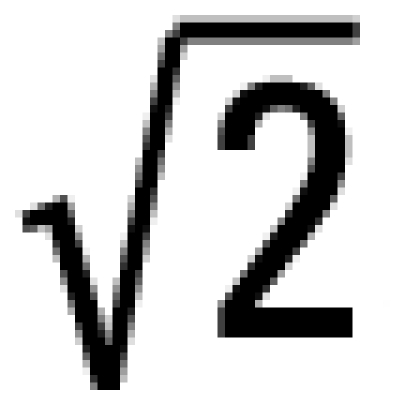
. Congeners are identified according to the International Union for Pure and Applied Chemistry (IUPAC) nomenclature.

b Median LOD: median of sample specific LOD for each congener.

**Table 3:** Predictors of log total serum concentration PCDD/PCDF/C-PCB (pg/g lipid) concentrations among 8- to 9-year-old boys in the Russian Children’s Study.

	Adjusted regression coefficient^a^,^b^		
Predictor	Estimate	95% CI	*p*-Value
Age (years)	0.138	(0.106 to 0.171)	< 0.0001
BMI (kg/m^2^)	−0.020	(−0.027 to −0.013)	< 0.0001
Duration of breast-feeding (weeks)	0.001	(0.001 to 0.001)	<0.0001
Maximum parental education^c^	−0.038	(−0.065 to −0.010)	0.007
Residence in Chapaevsk (years)	0.003	(−0.002 to 0.008)	0.26
Mother ever employed at Khimprom	0.090	(0.020 to 0.160)	0.01
Mother’s local gardening	0.045	(0.012 to 0.078)	0.008
Current residence, distance from Khimprom			
< 2 km	0.124	(0.073 to 0.176)	< 0.0001
2–5 km	0.035	(−0.001 to 0.070)	0.06
> 5 km	Reference		
Any local eggs eaten	0.138	(0.097 to 0.179)	< 0.0001
Any local nonpoultry meat eaten	0.095	(0.026 to 0.163)	0.007
Any local poultry eaten	0.074	(0.014 to 0.134)	0.02
Any local dairy eaten	0.062	(0.029 to 0.095)	< 0.0001
Any local fish eaten	0.024	(−0.016 to 0.065)	0.24
Highest category of local fruit/vegetable eaten^d^	0.047	(−0.039 to 0.133)	0.29

a Adjusted for age, BMI, breast-feeding, parental education, residence in Chapaevsk, mother ever employed at Khimprom, mother’s local gardening, residential distance from Khimprom.

b Local foods separately included in multivariate model, adjusted for total consumption.

c Ordinal: reference level = secondary education or less, with higher levels of junior college/technical training and university graduate.

d Reference = lowest category of local consumption.

**Table 4:** Predictors of log total 2005 TEQs (PCDDs/PCDFs/C-PCBs/M-PCBs) (pg/g lipid) among 8- to 9-year-old boys in the Russian Children’s Study.

	Adjusted regression coefficient^a^,^b^		
Predictor	Estimate	95% CI	*p*-Value
Age (years)	0.102	(0.057 to 0.147)	< 0.0001
BMI (kg/m^2^)	−0.032	(−0.042 to −0.023)	< 0.0001
Duration of breast-feeding (weeks)	0.002	(0.002 to 0.003)	< 0.0001
Maximum parental education^c^	−0.034	(−0.072 to 0.004)	0.08
Residence in Chapaevsk (years)	0.008	(0.001 to 0.016)	0.03
Mother ever employed at Khimprom	0.112	(0.016 to 0.209)	0.02
Mother’s local gardening	0.091	(0.045 to 0.137)	< 0.0001
Current residence, distance from Khimprom			
< 2 km	0.212	(0.141 to 0.282)	< 0.0001
2–5 km	0.072	(0.022 to 0.122)	0.005
> 5 km	Reference		
Any local eggs eaten	0.215	(0.158 to 0.271)	< 0.0001
Any local nonpoultry meat eaten	0.150	(0.056 to 0.244)	0.002
Any local poultry eaten	0.143	(0.060 to 0.225)	0.001
Any local dairy eaten	0.085	(0.038 to 0.131)	< 0.0001
Any local fish eaten	0.076	(0.020 to 0.133)	0.008
Highest category of local fruit/vegetable eaten^d^	0.140	(0.021 to 0.259)	0.02

a Adjusted for age, BMI, breast-feeding, parental education, residence in Chapaevsk, mother’s employment at Khimprom, mother’s local gardening, residential distance from Khimprom.

b Local foods separately included in multivariate model, adjusted for total consumption.

c Ordinal: reference level = secondary education or less, with higher levels of junior college/technical training and university graduate.

d Reference = lowest category of local consumption.

**Table 5:** Predictors of log total serum concentration of PCBs (ng/g lipid) among 8- to 9-year-old boys in the Russian Children’s Study.

	Adjusted regression coefficient^a^,^b^		
Predictor	Estimate	95% CI	*p*-Value
Age (years)	0.177	(0.135 to 0.219)	< 0.0001
BMI (kg/m^2^)	−0.038	(−0.047 to −0.029)	< 0.0001
Duration of breastfeeding (weeks)	0.003	(0.002 to 0.003)	< 0.0001
Maximum parental education^c^	−0.018	(−0.054 to 0.017)	0.31
Residence in Chapaevsk (years)	0.008	(0.001 to 0.015)	0.03
Mother ever employed at Khimprom	0.101	(0.011 to 0.190)	0.05
Mother’s local gardening	0.081	(0.039 to 0.124)	< 0.0001
Current residence, distance from Khimprom			
< 2 km	0.101	(0.034 to 0.167)	0.003
2–5 km	0.021	(−0.025 to 0.067)	0.38
> 5 km	Reference		
Any local eggs eaten	0.160	(0.105 to 0.214)	< 0.0001
Any local nonpoultry meat eaten	0.088	(−0.001 to 0.176)	0.05
Any local poultry eaten	0.094	(0.016 to 0.171)	0.02
Any local dairy eaten	0.049	(0.005 to 0.092)	0.03
Any local fish eaten	0.070	(0.017 to 0.122)	0.01
Highest category of local fruit/vegetable eaten^d^	0.089	(−0.022 to 0.200)	0.12

a Adjusted for age, BMI, breastfeeding, parental education, residence in Chapaevsk, mother’s employment at Khimprom, mother’s local gardening, residential distance from Khimprom.

b Local foods separately included in multivariate model, adjusted for total consumption.

c Ordinal: reference level = secondary education or less, with higher levels of junior college/technical training and university graduate.

d Reference = lowest category of local consumption.

## References

[b1-ehp-117-1593] Abraham K, Knoll A, Ende M, Papke O, Helge H (1996). Intake, fecal excretion, and body burden of polychlorinated diben-zo-*p*-dioxins and dibenzofurans in breast-fed and formula-fed infants. Pediatr Res.

[b2-ehp-117-1593] Akhmedkhanov A, Revich B, Adibi JJ, Zeilert V, Masten SA, Patterson DG (2002). Characterization of dioxin exposure in residents of Chapaevsk, Russia. J Expo Anal Environ Epidemiol.

[b3-ehp-117-1593] Arsenescu V, Arsenescu RI, King V, Swanson H, Cassis LA (2008). Polychlorinated biphenyl-77 induces adipocyte differentiation and proinflammatory adipokines and promotes obesity and atherosclerosis. Environ Health Perspect.

[b4-ehp-117-1593] Baccarelli A, Giacomini SM, Corbetta C, Landi MT, Bonzini M, Consonni D (2008). Neonatal thyroid function in Seveso 25 years after maternal exposure to dioxin. PLoS Med.

[b5-ehp-117-1593] Baccarelli A, Pfeiffer R, Consonni D, Pesatori AC, Bonzini M, Patterson DG (2005). Handling of dioxin measurement data in the presence of non-detectable values: overview of available methods and their application in the Seveso chloracne study. Chemosphere.

[b6-ehp-117-1593] Bilau M, De Henauw S, Schroijen C, Bruckers L, Hond ED, Koppen G (2009). The relation between the estimated dietary intake of PCDD/Fs and levels in blood in a Flemish population (50–65 years). Environ Int.

[b7-ehp-117-1593] Charnley G, Doull J (2005). Human exposure to dioxins from food, 1999–2002. Food Chem Toxicol.

[b8-ehp-117-1593] Choi AL, Levy JI, Dockery DW, Ryan LM, Tolbert PE, Altshul LM (2006). Does living near a Superfund site contribute to higher polychlorinated biphenyl (PCB) exposure?. Environ Health Perspect.

[b9-ehp-117-1593] Cole TJ, Bellizzi MC, Flegal KM, Dietz WH (2000). Establishing a standard definition for child overweight and obesity worldwide: international survey. BMJ.

[b10-ehp-117-1593] Cole TJ, Flegal KM, Nicholls D, Jackson AA (2007). Body mass index cut offs to define thinness in children and adolescents: international survey. BMJ.

[b11-ehp-117-1593] Collins JJ, Bodner K, Burns CJ, Budinsky RA, Lamparski LL, Wilken M (2007). Body mass index and serum chlorinated dibenzo-*p*-dioxin and dibenzofuran levels. Chemosphere.

[b12-ehp-117-1593] Consonni D, Pesatori AC, Zocchetti C, Sindaco R, D’Oro LC, Rubagotti M (2008). Mortality in a population exposed to dioxin after the Seveso, Italy, accident in 1976:25 years of follow-up. Am J Epidemiol.

[b13-ehp-117-1593] Ecological Analytical Center (2007). Complex Assessment of the Environment for Dioxins and other Pollutants in Chapaevsk, Samara Region [in Russian]. Final report.

[b14-ehp-117-1593] Fernandez MA, Gomara B, Bordajandi LR, Herrero L, Abad E, Abalos M (2004). Dietary intakes of polychlorinated dibenzo-*p*-dioxins, dibenzofurans and dioxin-like polychlorinated biphenyls in Spain. Food Addit Contam.

[b15-ehp-117-1593] Fürst P (2006). Dioxins, polychlorinated biphenyls and other organohalogen compounds in human milk. Levels, correlations, trends and exposure through breastfeeding. Mol Nutr Food Res.

[b16-ehp-117-1593] Hauser R, Williams P, Altshul L, Korrick S, Peeples L, Patterson DG (2005). Predictors of serum dioxin levels among adolescent boys in Chapaevsk, Russia: a cross-sectional pilot study. Environ Health.

[b17-ehp-117-1593] Hayward DG, Bolger PM (2005). Tetrachlorodibenzo-*p*-dioxin in baby food made from chicken produced before and after the termination of ball clay use in chicken feed in the United States. Environ Res.

[b18-ehp-117-1593] Heudorf U, Angerer J, Drexler H (2002). Polychlorinated biphenyls in the blood plasma: current exposure of the population in Germany. Rev Environ Health.

[b19-ehp-117-1593] Humblet O, Birnbaum L, Rimm E, Mittleman MA, Hauser R (2008). Dioxins and cardiovascular disease mortality. Environ Health Perspect.

[b20-ehp-117-1593] Kerger BD, Leung HW, Scott PK, Paustenbach DJ (2007). Refinements on the age-dependent half-life model for estimating child body burdens of polychlorodibenzodioxins and dibenzofurans. Chemosphere.

[b21-ehp-117-1593] Kerger BD, Leung HW, Scott PK, Paustenbach DJ, Needham LL, Patterson DG (2006). Age- and concentration-dependent elimination half-life of 2,3,7,8-tetrachlorodiben-zo-*p*-dioxin in Seveso children. Environ Health Perspect.

[b22-ehp-117-1593] Kreuzer PE, Csanady GA, Baur C, Kessler W, Papke O, Greim H (1997). 2,3,7,8-Tetrachlorodibenzo-*p*-dioxin (TCDD) and congeners in infants. A toxicokinetic model of human lifetime body burden by TCDD with special emphasis on its uptake by nutrition. Arch Toxicol.

[b23-ehp-117-1593] Landi MT, Consonni D, Patterson DG, Needham LL, Lucier G, Brambilla P (1998). 2,3,7,8-Tetrachlorodibenzo-*p*-di-oxin plasma levels in Seveso 20 years after the accident. Environ Health Perspect.

[b24-ehp-117-1593] Lee MM, Sergeyev O, Williams P, Korrick S, Zeilert V, Revich B (2003). Physical growth and sexual maturation of boys in Chapaevsk, Russia. J Pediatr Endocrinol Metab.

[b25-ehp-117-1593] Link B, Gabrio T, Zoellner I, Piechotowski I, Paepke O, Herrmann T (2005). Biomonitoring of persistent organochlorine pesticides, PCDD/PCDFs and dioxin-like PCBs in blood of children from South West Germany (Baden-Wuerttemberg) from 1993 to 2003. Chemosphere.

[b26-ehp-117-1593] Longnecker MP, Michalek JE (2000). Serum dioxin level in relation to diabetes mellitus among Air Force veterans with background levels of exposure. Epidemiology.

[b27-ehp-117-1593] Maervoet J, Vermeir G, Covaci A, Van Larebeke N, Koppen G, Schoeters G (2007). Association of thyroid hormone concentrations with levels of organochlorine compounds in cord blood of neonates. Environ Health Perspect.

[b28-ehp-117-1593] Martinchik AN, Baturin AK, Baeva VS, Feoktistova AI, Piatnitskaia IN, Azizbekian GA (1998). Development of a method of studying actual nutrition according to analysis of the frequency of consumption of food products: creation of a questionnaire and general evaluation of the reliability of the method [in Russian]. Vopr Pitan.

[b29-ehp-117-1593] Michalek JE, Pavuk M (2008). Diabetes and cancer in veterans of Operation Ranch Hand after adjustment for calendar period, days of spraying, and time spent in southeast Asia. J Occup Environ Med.

[b30-ehp-117-1593] Michalek JE, Pirkle JL, Caudill SP, Tripathi RC, Patterson DG, Needham LL (1996). Pharmacokinetics of TCDD in veterans of Operation Ranch Hand: 10-year follow-up. J Toxicol Environ Health.

[b31-ehp-117-1593] Mocarelli P, Gerthoux PM, Patterson DG, Milani S, Limonta G, Bertona M (2008). Dioxin exposure, from infancy through puberty, produces endocrine disruption and affects human semen quality. Environ Health Perspect.

[b32-ehp-117-1593] Nagayama J, Tsuji H, Iida T, Nakagawa R, Matsueda T, Hirakawa H (2007). Immunologic effects of perinatal exposure to dioxins, PCBs and organochlorine pesticides in Japanese infants. Chemosphere.

[b33-ehp-117-1593] Patandin S, Dagnelie PC, Mulder PG, Op de Coul E, van der Veen JE, Weisglas-Kuperus N (1999). Dietary exposure to polychlorinated biphenyls and dioxins from infancy until adulthood: a comparison between breast-feeding, toddler, and long-term exposure. Environ Health Perspect.

[b34-ehp-117-1593] Patterson DG, Hampton L, Lapeza CR, Belser WT, Green V, Alexander L (1987). High-resolution gas chromatographic/high-resolution mass spectrometric analysis of human serum on a whole-weight and lipid basis for 2,3,7,8-tetrachlorodibenzo-*p*-dioxin. Anal Chem.

[b35-ehp-117-1593] Patterson DG, Turner WE, Caudill SP, Needham LL (2008). Total TEQ reference range (PCDDs, PCDFs, cPCBs, mono-PCBs) for the US population 2001–2002. Chemosphere.

[b36-ehp-117-1593] Petrik J, Drobna B, Pavuk M, Jursa S, Wimmerova S, Chovancova J (2006). Serum PCBs and organochlorine pesticides in Slovakia: age, gender, and residence as determinants of organochlorine concentrations. Chemosphere.

[b37-ehp-117-1593] Phillips DL, Pirkle JL, Burse VW, Bernert JT, Henderson LO, Needham LL (1989). Chlorinated hydrocarbon levels in human serum: effects of fasting and feeding. Arch Environ Contam Toxicol.

[b38-ehp-117-1593] Revich B, Aksel E, Dvoirin V, Kolbeneva L, Pervunina R (1996). Dioxin in the environment of Chapaevsk (Russia), health of its population. Organohalogen Compounds.

[b39-ehp-117-1593] Revich B, Aksel E, Ushakova T, Ivanova I, Zhuchenko N, Klyuev N (2001). Dioxin exposure and public health in Chapaevsk, Russia. Chemosphere.

[b40-ehp-117-1593] Revich B, Brodsky E, Sotskov Y (1999). Dioxin in environmental, blood, breast milk, cow milk in Chapaevsk town. Organohalogen Compounds.

[b41-ehp-117-1593] Rockett HR, Breitenbach M, Frazier AL, Witschi J, Wolf AM, Field AE (1997). Validation of a youth/adolescent food frequency questionnaire. Prev Med.

[b42-ehp-117-1593] Ryan JJ, Schecter A (2000). Exposure of Russian phenoxy herbicide producers to dioxins. J Occup Environ Med.

[b43-ehp-117-1593] Schecter A, Cramer P, Boggess K, Stanley J, Papke O, Olson J (2001). Intake of dioxins and related compounds from food in the U.S. population. J Toxicol Environ Health A.

[b44-ehp-117-1593] Schecter A, Quynh HT, Pavuk M, Papke O, Malisch R, Constable JD (2003). Food as a source of dioxin exposure in the residents of Bien Hoa City, Vietnam. J Occup Environ Med.

[b45-ehp-117-1593] Schoeters G, Hoogenboom R (2006). Contamination of free-range chicken eggs with dioxins and dioxin-like polychlorinated biphenyls. Mol Nutr Food Res.

[b46-ehp-117-1593] Sergeyev O, Saharov I, Shelepchikov AA, Revich B, Sotskov Y, Brodsky E (2007). Levels of PCDDs/PCDFs in the environment and food after 3 years of full plant inactivity, Chapaevsk, Russia. Organohalogen Compounds.

[b47-ehp-117-1593] Sergeyev O, Shelepchikov AA, Denisova T, Revich B, Saharov I, Sotskov Y (2008). POPs in human milk in Chapaevsk, Russia, five years following cessation of chemical manufacturing and decade of remediation program, pilot study. Organohalogen Compounds.

[b48-ehp-117-1593] Shelepchikov A, Sergeyev O, Revich B, Saharov I, Sotskov Y, Brodsky E (2008). Chlorine industry in the former USSR, Chapaevsk, Russia. Organohalogen Compounds.

[b49-ehp-117-1593] Shelepchikov AA, Revich BA, Feshin DB, Brodsky S, Zilnikov VG, Sergeyev O (2006). Contamination of chicken eggs from different Russian regions by PCBs and chlorinated pesticides. Organohalogen Compounds.

[b50-ehp-117-1593] Steenland K, Piacitelli L, Deddens J, Fingerhut M, Chang LI (1999). Cancer, heart disease, and diabetes in workers exposed to 2,3,7,8-tetrachlorodibenzo-*p*-dioxin. J Natl Cancer Inst.

[b51-ehp-117-1593] Turner W, DiPietro E, Lapeza C, Green V, Gill J, Patterson DG (1997). A fast universal automated cleanup system for the isotope-dilution high-resolution mass spectrometric analysis of PCDDs, PCDFs, coplanar PCBs, PCB congeners, and persistent pesticides from the same serum sample. Organohalogen Compounds.

[b52-ehp-117-1593] Van den Berg M, Birnbaum LS, Denison M, De Vito M, Farland W, Feeley M (2006). The 2005 World Health Organization reevaluation of human and mammalian toxic equivalency factors for dioxins and dioxin-like compounds. Toxicol Sci.

[b53-ehp-117-1593] Wang SL, Tsai PC, Yang CY, Leon Guo Y (2008). Increased risk of diabetes and polychlorinated biphenyls and dioxins: a 24-year follow-up study of the Yucheng cohort. Diabetes Care.

[b54-ehp-117-1593] WHO (2007). Growth Reference Data for 5–19 Years.

[b55-ehp-117-1593] Wigle DT, Arbuckle TE, Turner MC, Berube A, Yang Q, Liu S (2008). Epidemiologic evidence of relationships between reproductive and child health outcomes and environmental chemical contaminants. J Toxicol Environ Health B Crit Rev.

[b56-ehp-117-1593] Wolff MS, Britton JA, Boguski L, Hochman S, Maloney N, Serra N (2008). Environmental exposures and puberty in inner-city girls. Environ Res.

